# Use of Genetically Encoded Calcium Indicators (GECIs) Combined with Advanced Motion Tracking Techniques to Examine the Behavior of Neurons and Glia in the Enteric Nervous System of the Intact Murine Colon

**DOI:** 10.3389/fncel.2015.00436

**Published:** 2015-11-10

**Authors:** Grant W. Hennig, Thomas W. Gould, Sang Don Koh, Robert D. Corrigan, Dante J. Heredia, Matthew C. Shonnard, Terence K. Smith

**Affiliations:** Department of Physiology and Cell Biology, University of Nevada School of MedicineReno, NV, USA

**Keywords:** GECIs, myenteric plexus, submucous plexus, CMMC, colon, calcium

## Abstract

Genetically encoded Ca^2+^ indicators (GECIs) have been used extensively in many body systems to detect Ca^2+^ transients associated with neuronal activity. Their adoption in enteric neurobiology has been slower, although they offer many advantages in terms of selectivity, signal-to-noise and non-invasiveness. Our aims were to utilize a number of cell-specific promoters to express the Ca^2+^ indicator GCaMP3 in different classes of neurons and glia to determine their effectiveness in measuring activity in enteric neural networks during colonic motor behaviors. We bred several GCaMP3 mice: (1) Wnt1-GCaMP3, all enteric neurons and glia; (2) GFAP-GCaMP3, enteric glia; (3) nNOS-GaMP3, enteric nitrergic neurons; and (4) ChAT-GCaMP3, enteric cholinergic neurons. These mice allowed us to study the behavior of the enteric neurons in the intact colon maintained at a physiological temperature, especially during the colonic migrating motor complex (CMMC), using low power Ca^2+^ imaging. In this preliminary study, we observed neuronal and glial cell Ca^2+^ transients in specific cells in both the myenteric and submucous plexus in all of the transgenic mice variants. The number of cells that could be simultaneously imaged at low power (100–1000 active cells) through the undissected gut required advanced motion tracking and analysis routines. The pattern of Ca^2+^ transients in myenteric neurons showed significant differences in response to spontaneous, oral or anal stimulation. Brief anal elongation or mucosal stimulation, which evokes a CMMC, were the most effective stimuli and elicited a powerful synchronized and prolonged burst of Ca^2+^ transients in many myenteric neurons, especially when compared with the same neurons during a spontaneous CMMC. In contrast, oral elongation, which normally inhibits CMMCs, appeared to suppress Ca^2+^ transients in some of the neurons active during a spontaneous or an anally evoked CMMC. The activity in glial networks appeared to follow neural activity but continued long after neural activity had waned. With these new tools an unprecedented level of detail can be recorded from the enteric nervous system (ENS) with minimal manipulation of tissue. These techniques can be extended in order to better understand the roles of particular enteric neurons and glia during normal and disordered motility.

## Introduction

The function of neural networks, and their role in the generation of complex patterns of motor behavior in the gut is inherently difficult to study because of the large number of interacting neurons that have emergent properties that can’t readily be predicted from recording from individual neurons (Bayguinov et al., 2010a,b,c).

The enteric nervous system (ENS) in the bowel consists of an intricate system of layered and interconnected ganglionated neural networks that regulate motility, secretion and blood flow (Lomax and Furness, 2000; Furness, 2012; Okamoto et al., 2014; Smith et al., 2015). The largest and best known layer of neurons, the myenteric plexus, located between the longitudinal and circular muscle layers, is a two-dimensional sheet of interconnected ganglia that generates and coordinates many aspects of motor behavior of the colon (Bayguinov et al., 2010a,b). In the large bowel, most myenteric ganglia contain many different functional classes of neurons: sensory neurons, several ascending and descending interneurons, as well as excitatory and inhibitory motor neurons (Lomax and Furness, 2000; Smith et al., 2007).

Myenteric neurons are surrounded and embedded in a network of glial cells and their processes, which were largely thought to be the “glue” that held ganglia together (Gulbransen and Sharkey, 2012). Glia cells respond to a variety of neurotransmitter agonists (e.g., ACh, ATP, 5-HT, NK2, substance P) by releasing intracellular stores of Ca^2+^, and appear to be innervated or in close proximity to nerve varicosities (e.g., including seroternergic and sympathetic nerves: Broadhead et al., 2012; Gulbransen and Sharkey, 2009, 2012; Boesmans et al., 2013a; Okamoto et al., 2014). Furthermore, glial cells can display prolonged Ca^2+^ responses compared to myenteric neurons, peaking some ~40 s following neural reflex stimulation (Broadhead et al., 2012). This is more prolonged than glial Ca^2+^ responses observed after addition of purinergic, 5-HT and ACh agonists (Gulbransen and Sharkey, 2009; Boesmans et al., 2013a; McClain et al., 2014). Ca^2+^ waves in glial processes, which surround neurons, appear to summate in their soma where they possibly release substances such as prostaglandins, nitric oxide or purines that either help terminate or enhance the excitability of specific neurons (MacEachern et al., 2011; Broadhead et al., 2012; Heredia et al., 2012; Smith et al., 2015). These observations support the notion that enteric glia, similar to those in the peripheral and central nervous systems, may not just sense, but also modulate neurotransmission (Robitaille, 1998).

Using genetically-encoded calcium indicators such as GCaMP3 (Tian et al., 2009; Wilms and Häusser, 2009; Akerboom et al., 2012; Yamada and Mikoshiba, 2012; Zariwala et al., 2012; Sun et al., 2013) expressed in various enteric neurons and glia (Boesmans et al., 2015; Foong et al., 2015), we show here that the behavior of these cells and their role in colonic motor patterns can be readily analyzed in the undissected isolated colon using low-power imaging of GCaMP3. The specificity and quality of labeling of neurons and glia with GCaMP3 allows a much better understanding of how motor behaviors emerge from the activity of individual neurons in the gut both *in situ* and *in vivo*.

## Methods

### Generation of Specific GCaMP3 Mice

All mice (C57BL/6 background of either sex) were housed in a transgenic animal facility under pathogen-free conditions on a 12 h light/dark cycle with food and water *ad libitum*. The following mice were generated for this study: *Wnt1-Cre; Rosa26-Lox-STOP-Lox-GCaMP3* mice (GCaMP3 targeted to neural crest derivatives, including enteric neurons and glia via Wnt1-Cre transgenic mice; Jax #009107; heretofore referred to as Wnt1-GCaMP3), *GFAP-Cre; Rosa26-Lox-STOP-Lox-GCaMP3* (GCaMP3 targeted to glia via mice expressing Cre from the human GFAP promoter; Jax #004600; heretofore referred to as GFAP-GCaMP3), *ChAT-Cre; Rosa26-Lox-STOP-Lox-GCaMP3* (GCaMP3 targeted to cholinergic neurons via ChAT-Cre BAC transgenic mice; MMRRC #37336; heretofore referred to as ChAT-GCaMP3) and *nNOS-CreER; Rosa26-Lox-STOP-Lox-GCaMP3* (GCaMP3 targeted to nitrergic neurons via nNOS-CreER transgenic mice; Jax #014541; heretofore referred to as nNOS-GCaMP3). Mice expressing a conditional (i.e., Cre-dependent) allele of GCaMP3 in the Rosa26 locus (*Rosa26-Lox-STOP-Lox-GCaMP3*) were described previously (Jax # 014538; Zariwala et al., 2012). nNOS-GCaMP3 mice were given intraperitoneal injections of tamoxifen (TM) between the ages of 4–6 weeks in order to activate CreER and hence GCaMP3 expression. TM was first dissolved in 100% ethanol to a concentration of 100 μg/μl by vortexing for 15 min. Then this TM stock solution was further dissolved 1:5 in safflower oil, vortexed for 20 min and sonicated for 30 min immediately prior to injection. 100 μl of this working TM solution (2 mg) was injected intraperitoneally for three consecutive days, and animals were sacrificed 5–10 days after the last injection.

These procedures were in accordance with National Institutes of Health guidelines for the care and use of laboratory animals and approved by the Animal Ethics Committee at the University of Nevada, Reno.

### Tissue Preparation

**Figure 1:**
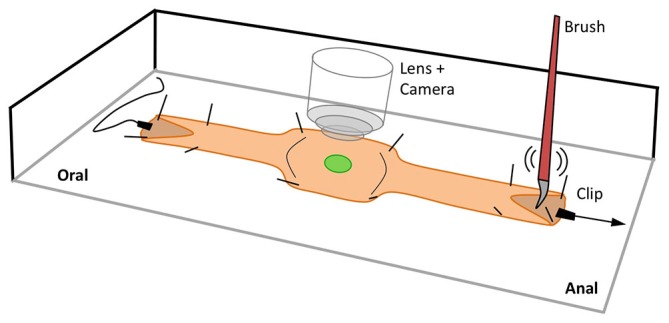
**Schematic of the experimental setup used to record Ca^2+^ activity in targeted cells in the isolated intact mouse colon.** The ends of the preparation were slit and pinned open to allow for mucosal stimulation using a brush and/or longitudinal stretch stimulation using clips. In the middle of the preparation where recordings were taken, the colon was stabilized by pinning through the outer edges.

Mice were euthanized by inhalation of a 5% concentration of isoflurane, followed by cervical dislocation. A ventral midline incision was made and the whole colon (proximal colon and distal colon) was carefully excised. The entire large intestine was removed from the transgenic mice and the mesentery trimmed and pinned to the bottom of a 30 mL Sylgard-lined (Dow Corning Corp., Midland, MI, USA) organ bath in 4°C oxygenated Krebs Ringer Buffer solution (KRB, concentrations in mM: NaCl: 120.35; KCl: 5.9; NaHCO_3_: 15.5; MgCl_2_: 1.2; NaH_2_PO_4_: 1.2; glucose: 11.5; CaCl_2_: 2.5; pH 7.4) oxygenated with 97% O_2_ and 3% CO_2_. The ends of the colon (~10 length) were cut open along the mesenteric border and pinned flat, mucosal side uppermost, to allow for brush stimulation of the mucosa to evoke neural reflexes that elicit colonic migrating motor complexes (CMMCs; Bayguinov et al., 2010a,b; Heredia et al., 2009). In some preparations the ends of the colon were attached to a frog heart clip to allow the colon to be briefly elongated in a localized pinned region by up to 10% of its length in order to evoke polarized descending inhibitory and excitatory reflexes triggered by colonic elongation as shown in Figure 1. Oral elongation (longitudinal stretch at the oral end of the preparation) inhibits CMMCs, whereas anal elongation (longitudinal stretch at the anal end of the preparation) generates a single CMMC (Heredia et al., 2010). The preparation was allowed to equilibrate for ~1 h as the bath gradually reached the recording temperature of 36–37°C. Most of our Ca^2+^ imaging was mainly restricted to the mid colon region approximately in the middle of the whole colon (~40 long) so that we could compare activity in GCaMP3 cells with that obtained previously with Ca^2+^ dyes (Bayguinov et al., 2010a,b, 2012; Heredia et al., 2010; Broadhead et al., 2012).

### Image Acquisition

Ca^2+^ imaging recordings were performed on an Olympus BX51WI upright fluorescence microscope using 10 and 20× Fluor water immersion lenses (Olympus, Center Valley, PA, USA). The tissue was excited at 488 nm, using a X-Cite series 120Q (Lumen Dynamics, Ontario, Canada) and a modified GFP dichroic cube (excitation 488 nm; emission 543 nm; Chroma Technology Corp., Bellows Falls, VT, USA). Movies were captured with an Andor iXon +897 EMCCD camera (normal recording ~2000 frames @ 32.4 Hz) using Andor Solis 4.14 software (Andor Technology, Belfast, UK). Solis (16-bit, tif) files were analyzed on a MacPro desktop computer (Apple Inc., Cupertino, CA, USA) using in-house analysis software (Volumetry G8c, G. W. Hennig).

### Drugs Used

Hexamethonium bromide was purchased from Sigma-Aldrich (St. Louis, MO, USA).

## Results

Ca^2+^-induced fluorescence in enteric neurons and glia expressing GCaMP3 was significantly brighter with better signal-to-noise ratio compared to Ca^2+^ dyes (e.g., Fluo-4, see Bayguinov et al., 2010a,b; Okamoto et al., 2012) such that no dissection of the tissue was needed. Labeled cells were observed throughout the entire gut. By focusing through the serosa and longitudinal muscle, we could easily visualize the myenteric plexus without confocal imaging. This allowed, in tandem with better cameras, imaging of much greater fields of view while still being able to resolve individual cells and their Ca^2+^ transients (10×). At low magnifications, Ca^2+^ transients in cell bodies could be measured, however, activity in fibers and varicosities could not be resolved at low power but could be resolved at higher power (Bayguinov et al., 2012). Ca^2+^ transients in cells in the myenteric plexus or submucous plexus (after focusing even deeper though the underlying circular muscle) were easily resolved without the need to dissect of any of the outer layers of the colon. The results presented below are produced from a representative subset of the animals tested during the development of the transgenic mice variants, and illustrate the Ca^2+^ responses in each of the targeted cells.

### Modified Image Analysis Suitable for GCaMP3 Expressing Cells

An obvious benefit of using GCaMP3 expressed in cells is that imaging can be performed without dissection, thus better preserving circumferential pathways and motor responses during a CMMC. However, without dissecting and stretching preparations flat (Bayguinov et al., 2010a), we were less physically able to restrain movements. Similarly, due to the absence of non-specific contamination of the surface of the preparation common with Ca^2+^ dyes, we were unable to utilize motion tracking routines that performed frame-by-frame comparisons using the pattern of static fluorescence. In this section, we demonstrate modified motion tracking routines, suitable for structures that display dynamic changes in intensity and demonstrate distortion correction rather than displacement correction to compensate for non-uniform contraction and relaxation.

### Automated Nuclei Tracking

**Figure 2:**
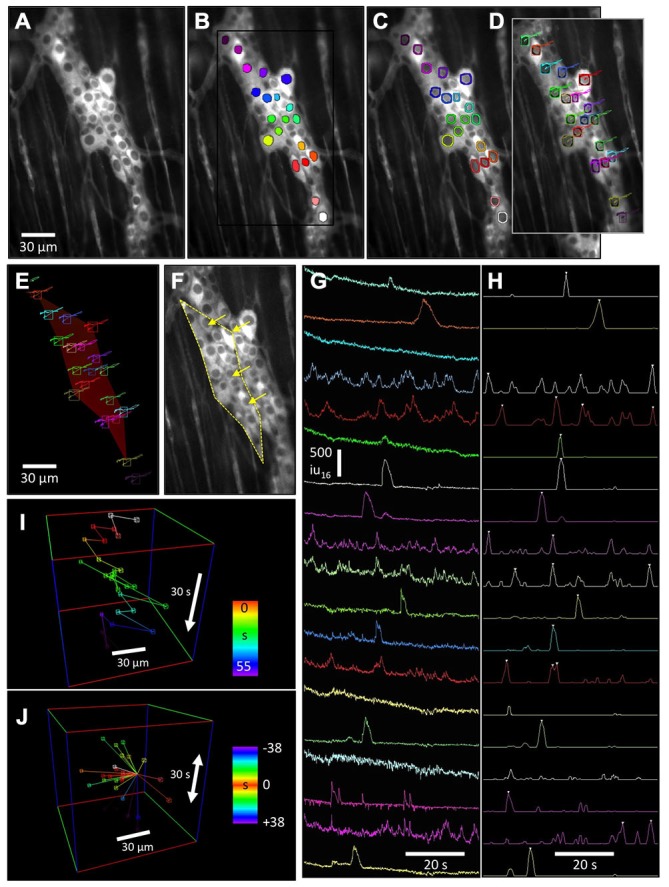
**Advanced motion tracking and analysis of cells labeled with GCaMP3 in myenteric ganglia.** The lack of non-fluorescing landmarks in tissues prevented the use of tracking routines based on the shape and intensity pattern of a reference region. Instead, to stabilize motion associated with CMMCs, the relatively darker nuclei (vs. cytoplasm) of these cells were used to generate ovoid “nuclear” particles. **(A)** Fluorescence image of a myenteric ganglion from a Wnt1-Cre-GCaMP3 mouse and **(B)** the same image showing these particles. **(C)** Nuclear particles were dilated to generate rings that were used to sample Ca^2+^ intensity changes in the cytoplasm around the nucleus. **(D)** Tracking of the nuclei was achieved using a radial differential routine around the edge of the “nuclear” particle, which maximized the sum of the sampled intensity slopes calculated from cytoplasm to the nucleus. The tracked trajectories are overlaid. **(E)** The tracked nuclei were used to generate movement vector maps (red color of reference polygon represents a mostly longitudinal displacement) which, when reversed, could be applied to the original movie to stabilize distortion to the reference shape **(F)** (arrows show direction and magnitude of the distortion correction), or used to calculate longitudinal and circular micro-motions of the preparation. **(G)** Plots of Ca^2+^ intensity for each neuron were then used to identify Ca^2+^ transients based on the upstroke slope and duration of each Ca^2+^ transient to generate an event sequence **(H)** (white triangles). The distance and angle between neurons that fired sequentially could be represented as **(I)** a 3D event sequence path plot, or **(J)** the time and position of firing events around a particular reference cell could represented.

The dark nuclei in cells expressing GCaMP3 were used as reference points since they had a relatively uniform size and shape. Briefly, a quiescent period (100–200 ms) in the recording was averaged to reduce noise (Figure 2A), then a threshold was ramped through the entire intensity range and particles generated for those areas matching a criteria of size and circularity of a nucleus (*area* = 25–50 μm^2^, radius_perimeter_:radius_area_ = 0.95–1.4). These “nuclear” particles were saved as a particle mask used for tracking (Figure 2B) and a separate particle mask was derived from the “nuclear” particles by performing a dilate routine (2 pixels) and removing the original “nuclear” particles. These peri-nuclear “rings” were used to extract changes in Ca^2+^ intensity from the cytoplasm of cells at a consistent radius around the nucleus (Figure 2C). Any manual corrections were done on the particle masks. ROIs were automatically created using bounding rectangles of individual particles and the ROIs and the “nuclear” particles were transferred to the original recording. Instead of using the absolute intensity value of the nuclei in the movie which alters during activity in a cell, the differential intensity between the nucleus and cytoplasm (at points ±2 pixels either side of the edge of the bitmask) was calculated every 30° (12 radial sampling points per nuclei). The centroid point within a search range that returned the maximum sum of differential intensities was stored and represents the most centralized position of the nucleus. This tracking was extremely fast and could process 15 neurons in a 2000 frame movie (±5 pixel search grid) in less than a second. The trajectories of each nuclei were smoothed (±60 ms see Figure 2D).

### Distortion Vector Maps

The movement trajectories of tracked cell nuclei were saved and used to generate movement vector maps. Traditional motion-tracking routines calculate displacement and/or rotation of the whole field of view (FOV), but do not take into account differential rotation, contraction and elongation (distortion). To calculate distortion within an area (e.g., ganglia), a reference frame was chosen and a bounding polygon that encompassed the outer edges of multiple tracked points was generated (Figures 2E,F). Tessellation of the polygon into individual triangles using tracking points within the bounding polygon enabled better estimates of any compression/elongation within the bounding polygon. Bilinear interpolation was used to blend the relative movements between the 3 pairs of trajectories used to create each triangle, allowing either a color movement offset map (per pixel X [red] and Y [green] offsets, see Figure 2E) or vector map (per pixel angle and distance) to be constructed for every frame of the movie.

The movement vector maps were applied to the original movie allowing complete stabilization of displacement, rotation, compression and expansion (distortion) within the reference bounding polygon (Figure 2F). After distortion had been stabilized in an area, fixed ROIs or bitmasks could be applied to ganglia for calculation of spatio-temporal maps (ST maps) or other analyses without motion artifacts. As an added feature, the information stored in movement vector maps can be used to quantify differential circular and longitudinal displacement and contraction within the bounding polygon.

### Non-Biased Measurement of Cytoplasmic Ca^2+^ Transients

After successful tracking of cells within ganglia using “nuclear” particles, the previously calculated “ring” masks were transferred to the tracked ROIs in the original movie, replacing the original “nuclear” particles masks. Ca^2+^-induced intensity changes were then measured in cells under the “ring” (Figures 2D,G). Average Ca^2+^-induced intensity changes within the “rings” are shown in (Figure 2G) although other intensity measurements are possible using this method, including the maximal intensity changes and the radial uniformity of Ca^2+^-induced fluorescence within the ring.

### Ca^2+^ Transient Event Discrimination and Event Sequences

After traces of Ca^2+^-induced intensity changes were generated per cell, they were smoothed (±30 ms average) and a discriminator used to detect the upstroke velocity and amplitude of Ca^2+^ transients (3 s window: Figure 2H). The discriminated traces were smoothed (±0.5 s) and peaks identified above the half maximum amplitude level. The temporal position of each discriminated Ca^2+^ transient in each cell was stored as an event sequence that saved the ROI number, frame and classification structure of each cell (e.g., ganglia). This allowed the spatial and temporal characteristics of neuronal Ca^2+^ events to be analyzed as 3D representations to better illustrate the sequence of activation of cells during a CMMC. The event sequence path (Figure 2I) is a graphical representation of the order in which cells in a myenteric ganglion fired during a CMMC where time at which a cell fired during the recording is color-coded. Multiple stimulations using the same stimuli can be used to determine the stability in the order of Ca^2+^ events and associations between individual neurons during the sequence of activation. Relative event plots (Figure 2J) centers on a reference cell and generates space-time vectors to the position of other cells that fired before or after the reference cell.

### Responses of Neurons and Glia to Different Stimuli

**Figure 3:**
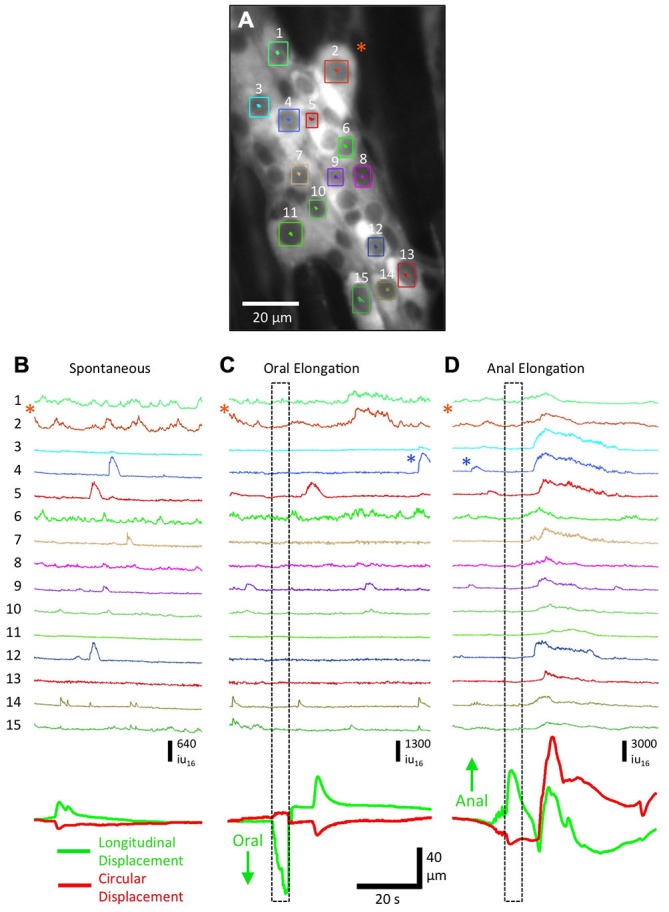
**Responses of myenteric ganglion cells to oral and anal elongation in Wnt1-GCaMP3 mice. (A)** Myenteric ganglion cells that remained in the FOV (during the contraction responses) and had sufficient nuclear edge contrast to enable tracking (labeled from 1–15). A neuron resembling a Dogiel Type II neuron is indicated by the orange asterisk. **(B)** During a spontaneous CMMC, a number of neurons showed Ca^2+^ transients, particularly ~10 after the onset of contraction. **(C)** During oral elongation a similar overall pattern was observed, except a few cells remained quiescent throughout the recording period and the resulting contraction was larger (see green longitudinal displacement and red circular displacement traces at bottom). **(D)** In marked contrast, anal elongation produced a robust Ca^2+^ response in all measured neurons, all within 1–2 s of each other following the elongation stimulus. Note the difference in the intensity scale bar between **(B,C)** and **(D)**. The Ca^2+^ transients indicated by the blue asterisks in **(C,D)** are of comparable amplitude.

**Figure 4:**
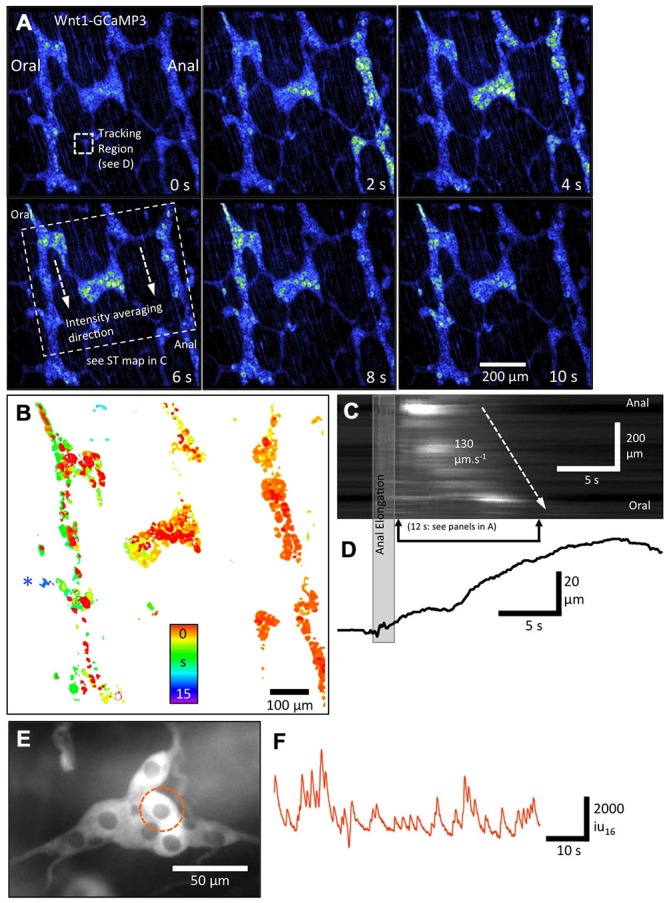
**Overall pattern of activation of all enteric neurons and glia in myenteric and submucous plexi using Wnt1-GCaMP3-labeled neural crest derived cells. (A)** Pseudo-colored sequence of images depicting the Ca^2+^-induced fluorescence of myenteric neurons and glia together during a CMMC evoked by anal elongation. Note the staggered activation of patches of neurons/glia in multiple ganglia with the rightmost ganglia displaying the most number of active neurons/glia initially followed, somewhat haphazardly by the middle and leftmost ganglia later on during the CMMC. **(B)** Temporal delay map was constructed by overlaying the area of thresholded Ca^2+^ transients at different time points and illustrates this apparent spread graphically over 15 s. The structure near the blue asterisk is a submucosal ganglia. **(C)** ST Map of the apparent propagation of activity between ganglia allowing an overall velocity of spread to be estimated. **(D)** A trace showing the time course of the distortion associated with the CMMC. **(E)** Higher-power image of an underlying smaller submucosal ganglia and **(F)** a plot of Ca^2+^-induced fluorescence in a selected cell in this ganglia.

**Figure 5:**
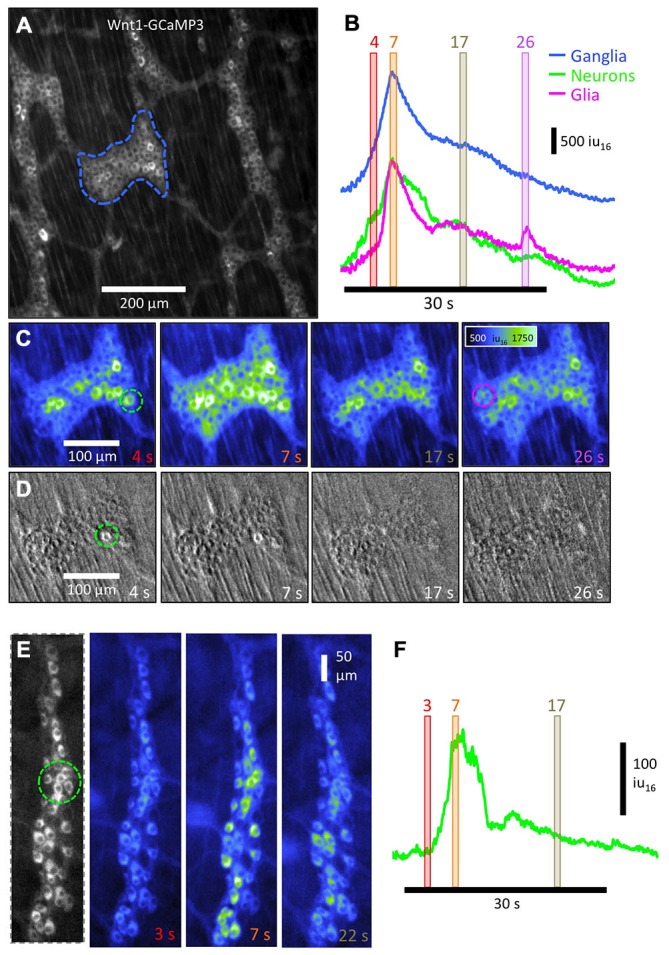
**Activation sequence of neurons and glia in colonic myenteric ganglia using Wnt1-GCaMP3 and ChAT- GCaMP3 labeled cells. (A)** Low- power image of multiple myenteric ganglia in which neurons and glia were both labeled with Wnt1-GCaMP3. **(B)** Overall measurement of Ca^2+^ activity from both cell types from the entire ganglia (blue trace) shows a build-up of Ca^2+^ over the first ~4 s of the CMMC followed by a biphasic decline in Ca^2+^ intensity over the following ~ 30 s. The CMMC was evoked by anal mucosal stimulation. Measuring selected regions in the ganglia that contained primarily neurons (green trace; see green dashed circle in C: 4 s) or glia (magenta trace: see magenta dashed circle in C: 26 s) based on their morphology illustrates how Ca^2+^ transients in neurons preceded glial activity by 1–2 s. This is better illustrated in the sequential images displayed in **(C)** where the mesh-like appearance of glia is visible in the 7 s panel, but not at 4 s panel. **(D)** To determine whether the number of neurons that fired affected the activation of glia, we added a submaximal dose of hexamethonium (5 μM) to compromise cholinergic nicotinic transmission between enteric neurons. Stimulation of the mucosa some distance away resulted in very few neurons becoming active at the site of recording and no observable glial response. Images were background subtracted to highlight those cells that fired after the stimulus. **(E)** To examine the neuronal response during the complex without concomitant glial activity, the activity in ChAT-GCaMP3 cholinergic neurons was analyzed. Images at 3 time points are displayed in pseudo-color and the time course of Ca^2+^ intensity in a small group of neurons **(F)** shows the characteristics of the increase of activity over the first 3–7 s, followed by a more rapid decline in the subsequent 7 s.

**Figure 6:**
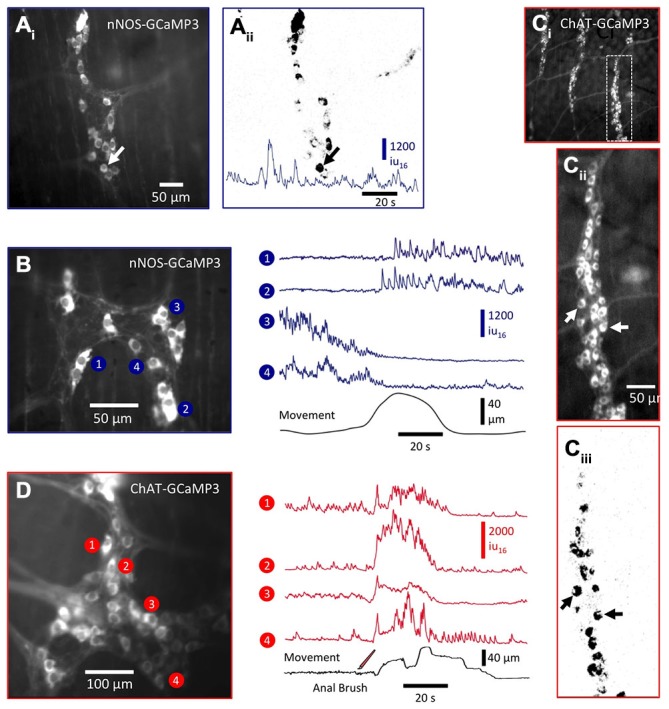
**Ca^2+^ activity in enteric neurons expressing nNOS- GCaMP3 and ChAT- GCaMP3. (A_i_)** Image of nNOS-GCaMP3 neurons in colonic myenteric ganglia and **(A_ii_)** an activity map of those neurons displaying spontaneous Ca^2+^ transients. A trace of spontaneous Ca^2+^ activity is shown at the bottom of the panel. **(B)** Image in the left panel shows another myenteric ganglia in which (right panel) the Ca^2+^ activity in selected neurons was plotted in relation to the distortion produced by a spontaneous CMMC (bottom trace). nNOS neurons displayed different responses during the onset of the CMMC: the top two traces show the activation of two formerly silent neurons, while the bottom two traces show the cessation/silencing of two formerly active neurons. These likely represent interneurons and inhibitory motor neurons respectively. **(C_i_)** Low-power image of ChAT-GCaMP3-expressing neurons in multiple colonic myenteric ganglia from which the rightmost ganglia was magnified **(C_ii_)** and an activity map created **(C_iii_)**. **(D)** Image in the left panel shows another ganglia in which the Ca^2+^ activity (right panel) in selected ChAT-GCaMP3 neurons was plotted. These neurons responded with excitatory responses during the initial phase of a CMMC evoked by mucosal stimulation (three strokes; see bottom trace) and likely represent a mixed population of IPANs, interneurons and excitatory motor neurons.

**Figure 7:**
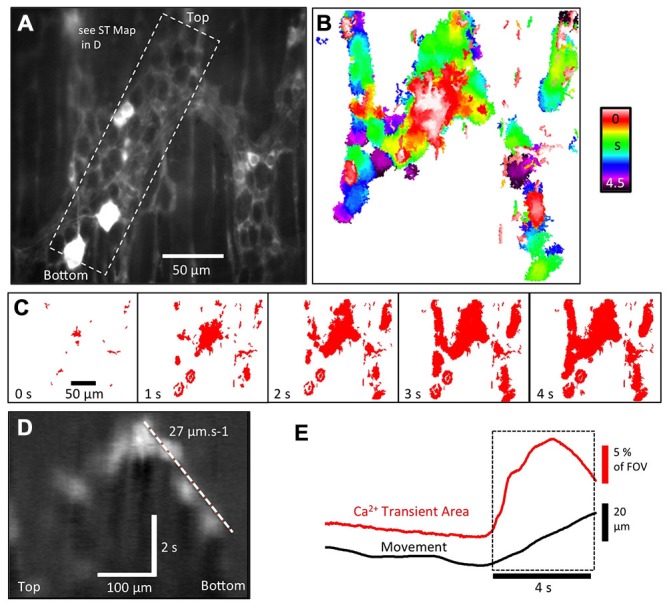
**Ca^2+^ activity in enteric glia expressing GFAP-GCaMP3 during the colonic CMMC. (A)** Fluorescence image of glial cell bodies and their extensive processes in a colonic myenteric ganglia. In addition to these glia, two neurons appear to express GCaMP3, as shown in the bottom left hand corner. **(B)** Colored temporal delay map illustrates that the activation of enteric glia and their processes during a CMMC was not uniform, but occurred over a period of 4.5 s in a rather “patchy” pattern. **(C)** Sequence of thresholded images used to generate the map in **(B)** revealed significant delays between the activation of different parts of the glial network in the same ganglia. **(D)** In some regions, the pattern of activation allowed the rate of spread to be calculated using ST maps (see dashed region in **A**). **(E)** The overall increase in GFAP-GCaMP3 Ca^2+^ intensity within a myenteric ganglia appears tightly correlated to the initiation of distortion (movement).

The lack of photobleaching of Ca^2+^-induced fluorescence in cells expressing GCaMP3 allowed experiments to be performed over many hours. This flexibility allowed a particular FOV to be imaged repeatedly during different stimulations or with drugs added. Advances in analysis allowed Ca^2+^ transients in individual cells within a ganglia could be extracted and compared. To illustrate the power of GCaMP3 in monitoring Ca^2+^ transients in neurons and glia we illustrate the power of the techniques by giving representative examples below in Figures 3–7. Anal elongation/mucosal stroking is usually a more effective stimulus to evoke a CMMC than oral elongation/mucosal stroking (Heredia et al., 2009, 2010, 2013; Bayguinov et al., 2010a). To ascertain the different responses in neurons and glia in a myenteric ganglia to oral and anal elongation, we used higher power imaging (20×) to track and record individual Ca^2+^ responses (Figure 3A). These evoked responses were compared to a spontaneous CMMC that occurred prior to the elongation stimuli. During a spontaneous CMMC (Figure 3B), a number of different patterns of Ca^2+^ transients were observed in individual cells: (i) complex Ca^2+^ transient activity (see Figure 3B: cells 1, 2 and 6) likely due to the variations in summation of ongoing Ca^2+^ transients; (ii) individual Ca^2+^ transients, likely associated with neuronal action potentials (see Figure 3B: cell 14); and (iii) long-duration Ca^2+^ transients (see Figure 3B: cells 4, 5 and 12) either representative of tetanic burst of action potentials in neurons or slower Ca^2+^ transients in glia. During the spontaneous CMMC, a cluster of activity in myenteric cells was observed ~10 s after the onset of contraction (see Figure 3B: bottom green and red traces representing longitudinal and circular displacement respectively) in eight cells, while three cells were quiescent throughout. During oral elongation, there was a ~5 s delay from the end of the stimulus to the start of contraction (see movement traces at bottom of Figure 3B). There was no apparent clustering of firing, although activity in three cells was increased (see Figure 3C: cells 2, 5 and 14). Two of the cells active during the spontaneous CMMC remained quiescent throughout the length of the recording after oral elongation (Figure 3C cells 7 and 12). In stark contrast to spontaneous or CMMCs evoked by oral elongation, anal elongation produced a considerable Ca^2+^ response in all measured cells clustered within 1–5 s of the cessation of the stimulus, and was coincident with a large circular and longitudinal contraction (Figure 3D: note the different scaling of traces. The Ca^2+^ transients next to the blue asterisks are equivalent in amplitude). This pronounced difference in response of myenteric neurons and glia to the polarity of elongation stimuli reveals the possible duality of the CMMC acting as both an ongoing “feed-forward” motor pattern that does not require local stimulation, as well as a response more similar to a peristaltic reflex with a strongly polarized, but long-lasting response to pronounced local stimulation.

### Propagation Patterns of Ca^2+^ Activity in Enteric Neurons and Glia During the CMMC in Wnt1-GCaMP3 Mice

To demonstrate how low-power (10×) imaging can resolve propagation of neural activity associated with a CMMC, the example in Figure 4 shows a FOV ~1 mm^2^ in size that encompasses three rows of ganglia. Lower-power imaging (10×) allowed single myenteric ganglion cells to be resolved and reduced the pixel shift associated with movement. Mucosal stimulation with a brush or a brief anal elongation were both effective stimuli to evoke a CMMC (Heredia et al., 2009, 2010, 2013). In Figure 4, anal elongation evoked a complex pattern of neuron and glial Ca^2+^ transients. Immediately after the stimulation, occasional neuron cell bodies scattered throughout the FOV became active (Figure 4A). The cells consisted of a variety of neurons, some of which were often large neurons that were ovoid in shape, similar to Dogiel Type 2 neurons (Figure 4A, 0 s; Bayguinov et al., 2010a; Okamoto et al., 2014). Over the following 10 s, there appeared to be a slow propagating wave of activity in which large numbers of myenteric ganglion cells displayed robust, prolonged Ca^2+^ transients going from right (anal) to left (oral) (see Figure 4A panels 0–10 s). This propagation can be better appreciated in the temporal delay map shown in Figure 4B where the time of peak Ca^2+^-induced fluorescence intensity was color-coded (spectrum). Significant delays in the activation of myenteric ganglion cells can be appreciated between, but also within individual ganglia (see middle ganglion in Figure 4B). The 10× lens has a large focal depth that allows for the significant delay in activation of cells in a submucosal ganglion located on a different focal plane on the left hand side of the FOV to be examined (Figure 4B: see blue asterisk next to ganglia). The speed of apparent propagation of the wave of activation of myenteric ganglion cells was calculated to be ~130 μms^−1^ from the ST map shown in Figure 4C. Distortion produced by muscular contraction began ~5 s after the initial activation of myenteric ganglion cells, with the most rapid phase of contraction occurring when the largest number of myenteric ganglion cells were active in the FOV. The distortion (contraction) continued to increase for some time (~5 s) after Ca^2+^ transients in these cells had dissipated (Figures 4C,D). These results demonstrate that an evoked CMMC involves a slow spreading but strong activation of most cells in myenteric ganglia, followed significantly later by the activation of cells in submucosal ganglia. Submucosal ganglion cells could be imaged in detail using a higher-power (20×) lens and displayed ongoing Ca^2+^ transients between complexes (Figures 4E,F).

### Temporal Activation Pattern of Neurons and Glia During a CMMC

When we examined the time courses of Ca^2+^ activity from myenteric neurons and glia (Figures 5A,B), it was clear that Ca^2+^ transients in glia cells occurred after the initial responding neurons (compare Figure 5C at 4 s with that at 7 s). Neurons were distinguished based on their larger size (nucleus and cytoplasmic area). The Ca^2+^ intensity of cells in a region of the ganglia that did not contain active neurons showed a 2–3 s delay compared to a region composed of mainly neurons (magenta and green circles in Figure 5C respectively). The combined Ca^2+^ intensity from the whole ganglion (Figure 5B blue line), neurons (Figure 2B green line) and glia (Figure 5B magenta line) all showed a biphasic response with a fast increase in overall Ca^2+^-induced fluorescence peaking at ~5 s after the first responders, followed by a fast decrease of Ca^2+^-induced fluorescence lasting another ~5 s. Following the faster rise and fall in overall Ca^2+^-induced fluorescence, there was a prolonged “plateau” or slowly declining phase lasting 20–30 s. During this phase, patches of glia cells were observed to fire sporadically as illustrated by the small peak in the glial trace (Figure 5B, 26 s).

To begin to examine the role of neuron-to-neuron communication on the responses of glia during a CMMC, we used a low dose of hexamethonium (5–10 μM, *n* = 4) to compromise cholinergic nicotinic transmission between neurons. This abolished nearly all neural activity, although a few neurons per ganglia were observed to display Ca^2+^ transients in response to mucosa or stretch stimulation (Figure 5D, green circle). However, no Ca^2+^ transients in glia were observed, suggesting either a critical mass of active neurons is required to activate glia, and/or that nicotinic transmission affects glia directly (Broadhead et al., 2012; Boesmans et al., 2013a; Petrov et al., 2014). To examine the response of neurons alone in more detail, we measured overall intensity of Ca^2+^ in a ganglion from a ChAT-GCaMP3 mouse. Similar to responses obtained from regions in Wnt1-GCaMP3 mice containing all classes of neurons, the time course of Ca^2+^-induced intensity changes from a group of ChAT-GCaMP3 neurons showed a similar biphasic response to anal elongation without glial contamination (Figures 5E,F).

### Ca^2+^ Activity in nNOS-GCaMP3 Neurons During the CMMC

Between CMMCs, a number nNOS-GCaMP3 neurons exhibited spontaneous Ca^2+^ transients (Figure 6: see arrowhead and trace in Figures 6Ai,ii,B and Figure 6B: neurons 3 and 4). During a spontaneous CMMC, a proportion of nNOS-GCaMP3 neurons reduced their Ca^2+^ transients (Figure 6B: neurons 3 and 4), consistent with these neurons being inhibitory motor neurons (Bayguinov et al., 2010a). Other nNOS-GCaMP3 neurons, which exhibited little spontaneous activity between CMMCs, displayed long, sustained trains of Ca^2+^ transients at the onset of the CMMC (Figure 6B: neurons 1 and 2). These neurons are likely to be a subclass of interneurons as has been reported previously using Ca^2+^ imaging via conventional Ca^2+^ dyes (Bayguinov et al., 2010a).

### Ca^2+^ Activity in ChAT-GCaMP3 Neurons During the CMMC

ChAT-GCaMP3 neurons were more numerous than nNOS-GCaMP3 neurons (compare Figures 6Ai,ii,B with Figures 6Ci,ii,D), which is not surprising since cholinergic neurons (~60% of all myenteric neurons) comprise many functionally different classes of neurons than nNOS neurons (Lomax and Furness, 2000; Hao et al., 2013). Some ChAT-GCaMP3 neurons exhibited ongoing Ca^2+^ transients between CMMCs and are likely to be mainly interneurons and inhibitory motor neurons (Figure 6D: neurons 1 and 2) as tonic inhibition dominates during this period (Dickson et al., 2010a,b). However, many ChAT-GCaMP3 neurons increased their activity during a CMMC (Figure 6D: neuron 4) and are likely to be excitatory motor neurons (Hao et al., 2013). Furthermore, by focusing deeper into the tissue through the circular muscle apparently separate classes of active nNOS and ChAT neurons could be observed in the submucous plexus and Henle’s plexus without removing the mucosa as was previously necessary (data not shown: see Okamoto et al., 2012, 2014).

These preliminary results mirror previous studies describing the behavior of NOS and ChAT neurons using Ca^2+^ dyes (Bayguinov et al., 2010a), but with a number of advantages including: (i) neurons could be visualized in preparations that were undissected (fully intact tubes with mucosa); (ii) no *post hoc* staining was needed to determine the class of neuron, as only those neurons with nNOS or ChAT expressed the Ca^2+^ indicator; (iii) the signal to noise of Ca^2+^-induced fluorescence in these cells enabled activity in these neurons to be observed in multiple ganglia simultaneously; and (iv) the lack of photobleaching and continual replenishment of GCaMP3 due to ongoing expression allowed activity in enteric neurons to be monitored for hours, allowing the effect of different drugs or stimulations to be observed in the same FOV. Morphological and functional differences in ChAT and nNOS enteric neurons allowed some differentiation of whether particular neurons were sensory (IPAN), ascending or descending inter- or motor neurons, but for unequivocal classification, live or *post hoc* labeling for known markers of these different functional classes of neurons will be necessary (Bayguinov et al., 2010a, 2012).

### Ca^2+^ Activity in GFAP-GCaMP3 Labeled Glia During the CMMC

We also studied the characteristic of activation of the myenteric glia network in GFAP-CaMP3 mice during the CMMC (Figure 7). On occasion, 1 or 2 large neurons, including Type II neurons, were labeled in GFAP-GCaMP3 mice (Figure 7A, see bottom two neurons). It has also been shown that a subpopulation of GFAP-positive progenitor cells can develop into neurons in the central nervous system (CNS; Casper and McCarthy, 2006) and in the ENS it has been shown that glial cells can gain a neurogenic potential in certain conditions (Joseph et al., 2011; Laranjeira et al., 2011). In any case, the labeling of these neurons was easy to exclude from analysis of glial response based on their size and background intensity. In GFAP-GCaMP3 mice, myenteric glial cell somas had a size of 14.0 ± 2.2 μm (long axis) and 6.9 ± 0.9 μm (short axis; 60 cells, *n* = 4) that is consistent with them being much smaller than neurons (Broadhead et al., 2012).

There was little activation of glial cells between CMMCs, although on occasions some random glial cell processes exhibited Ca^2+^ transients. However, the pattern of Ca^2+^ transients in the myenteric glial network following initiation of a CMMC was unique. Following anal elongation there was a slow spread of activity through the myenteric glia (Figures 7A–C) that can best be described as “stepped” or “patchy”. While we do not know the corresponding neural activity occurring within the myenteric ganglia, the propagation of activity was not smooth or coherent, nor could a wave front be resolved (see temporal delay map in Figure 7B). Whether there is bona fide propagation in the glial network (Figure 7D) or whether the activation of glia is dependent on adjacent neural activity remains to be determined. However, taking overall Ca^2+^ transient intensity throughout the ganglia shows a steady build-up of activity over 3–4 s during the initial phase of the CMMC (Figure 7E).

## Discussion

### Use of Ca^2+^ Indicators to Monitor Enteric Neurons and Glia

Calcium indicators such as Fluo3/4 have been used effectively to measure Ca^2+^ transients in enteric neurons, glia, pacemaker cells, smooth muscle and varicosities in order to monitor their activity during gut motor behaviors (Stevens et al., 1999; Spencer et al., 2006; Bayguinov et al., 2010a,b, 2012; Broadhead et al., 2012; Okamoto et al., 2012; Boesmans et al., 2013a,b; Smith et al., 2015). However, these indicators can be subject to photobleaching, dispersal from certain target cells, de-esterification issues and often need complex and time consuming dissection to target the dye to the cells of interest (Bayguinov et al., 2010a,b; Okamoto et al., 2012). While the kinetics of GCaMP3 are slower than Ca^2+^ indicator dyes, we chose this Ca^2+^ indicator as it has the brightest fluorescence (quantum yield) suitable for detecting Ca^2+^ transients when using low power imaging. The firing frequency of enteric neurons is usually less than 10 Hz (Mazzuoli and Schemann, 2012), and can likely resolve most Ca^2+^ transients generated by action potentials (Tian et al., 2009; Yamada and Mikoshiba, 2012).

In this study in which the GCaMP3 Ca^2+^ indicator was directly expressed in cells of interest, Ca^2+^-induced GCaMP3 fluorescence was bright enough that no dissection was required and visualization of either myenteric or submucosal neurons/glial cells could be readily made along the whole gut by focusing through the longitudinal or circular muscle layers respectively in an intact colon. Therefore, this method leaves the structure and connectivity of these plexuses and their relation to other cells intact, including the mucosa. Moreover, the shapes and relative sizes of different functional classes of neurons are well preserved (Lomax and Furness, 2000). We demonstrated that GCaMP3 appears to be good indicator of Ca^2+^ transient activity in enteric neurons and glia in the gut at physiological temperatures in Wnt1-GCaMP3 (all ENS neurons and glia), ChAT-GCaMP3 (enteric cholinergic neurons), nNOS-GCaMP3 (enteric nNOS neurons) and GFAP-GCaMP3 (enteric glia) mice both between and during the powerful, neurally-mediated CMMC (Bywater et al., 1989; Heredia et al., 2009; Bayguinov et al., 2010a).

Although there is some variation in the brightness of Ca^2+^ transients in different neurons and glia, this likely relates to variation in their Ca^2+^ handling ability. Importantly, myenteric neurons expressing GCaMP3 appear to respond normally to physiological stimuli such as mucosal stroking or elongation suggesting that the neural reflex circuitry is intact and not altered by the expression of the foreign protein or by the genetic manipulations necessary to generate these mice.

Also, by using ChAT-GCaMP3 and nNOS-GCaMP3 mice we can begin monitoring Ca^2+^ transient activity in broadly defined cholinergic and nitrergic neurons in myenteric and submucosal ganglia, without the necessity of time-consuming post-staining immunohistochemistry, as was previously necessary (see Bayguinov et al., 2010a,b, 2012; Broadhead et al., 2012; Okamoto et al., 2012). One caveat in utilizing such strategies, however, is that the expression of transgenes using promoters for cell-specific markers is not always matched to the endogenous expression pattern of the marker. For example, in a pair of studies using transgenic ChAT-Cre mice, it was found that transgene expression was not observed in all cells positive for ChAT-immunoreactivity (IR) and conversely that transgene expression was observed in a small percentage of cells negative for ChAT-IR (Hao et al., 2013; Erickson et al., 2014). The observation that expression of GFP in transgenic ChAT-GFP mice, as well as tdTomato expression in ChAT-Cre; Rosa26-Lox-STOP-Lox-tdTomato mice, are both found in ChAT-IR-negative cells (Erickson et al., 2014) supports the idea that this latter pattern represents ectopic transgene expression by ChAT-Cre mice rather than transient expression of endogenous ChAT, since transgenic ChAT-GFP mice do not lineage-trace cells the way ChAT-Cre; Rosa26-Lox-STOP-Lox-tdTomato mice do. In the future, these techniques will be able to be extended to identify the roles of subclasses of enteric neurons with different chemical markers.

### Characteristics of the CMMC Revealed by GCaMP3 Expressed in Different Classes of Enteric Neurons and Glia

The CMMC is a propagating contraction that is essentially a rhythmic peristaltic wave involving most cells across the colonic wall including myenteric and submucosal pacemaker cells and mucosal 5-HT release from enterochromaffin cells (Bayguinov et al., 2010a,b; Smith and Gershon, 2015; Smith et al., 2015). The CMMC appears to be equivalent to the high amplitude propagating contractions (HAPCs) observed in human colon (Smith et al., 2015).

Several ideas have been previously proposed for the generation and propagation of neurally-mediated motor complexes along the small and large intestine (see Smith et al., 2015). These ideas usually emphasize particular elements of the ENS as being more or less important for propulsive motor behaviors, e.g., a turning off of tonic inhibition, recurrent connections between IPANs, peristaltic reflexes and local reflexes generated by a pellet (Christensen et al., 1974; Bywater et al., 1989, 1998; Furness et al., 1996; Thomas et al., 2004; Heredia et al., 2009, 2013; Dinning et al., 2014). However, motor complexes likely invariably involve two main events: (1) generating an imbalance between inhibition and excitation to the smooth muscle to favor contraction and (2) propagating this activity along the gut. Because of gut movement, few groups have observed the interaction of all neurons in many ganglia covering a respectably-sized region of gut to verify their individual hypotheses (Bayguinov et al., 2010a,b; Okamoto et al., 2012).

What we deduce from our current studies using GCaMP3 mice is that CMMC generation likely involves the activation and inhibition of many types of neurons such as IPANs, interneurons, cholinergic motor neurons, inhibitory motor neurons, as well as glial cells. Immediately following a stimulus to activate a CMMC, a number of myenteric neurons, including likely Dogiel Type II neurons (IPANs), were activated; their activity lasts through much of the CMMC but is most pronounced in the first 10 s. However, subsequent to the initial activation of neurons and glia, there was a long lasting “plateau” or slowly decaying phase of activity over 20–30 s that is similar (albeit somewhat shorter) than the overall contractile responses occurring during a CMMC contraction (Dickson et al., 2010a). Furthermore, the apparent slow propagation of activation of neurons in adjacent ganglia in the myenteric plexus (~130 μms^−1^) may contribute to the slow propagation of the contraction associated with the CMMC (0.8s^−1^). The CMMC does not appear to be exclusively a simple peristaltic reflex, as described for the small intestine (Furness et al., 1996) that conducts rapidly through the myenteric plexus (Heredia et al., 2013), but is much slower, more elaborate and long-lasting—likely involving both a simple reflex activated immediately after the stimulus, followed by longer lasting complex interactions between neurons and glia.

The most effective stimuli for generating a CMMC was anal mucosal stimulation or anal elongation of the colon (Heredia et al., 2009, 2010) since these stimuli elicited a synchronized, prolonged and robust burst of Ca^2+^ transients compared to the activation of the same myenteric neurons observed during a spontaneous CMMC. Presumably this is because descending inhibitory nerve pathways are cut at the anal end of the colon, whereas ascending neural pathways are intact, although the point of origin of a spontaneous CMMC is difficult to detect (Spencer et al., 2005; Heredia et al., 2009, 2013; Bayguinov et al., 2010a).

The complexity of the neural circuitry underlying motor complexes is suggested by the fact that while a small percentage of neurons could be activated by stimuli in the presence of submaximal doses of hexamethonium (nicotinic antagonist), there was no CMMC-like contraction or glial response, indicating a critical number of neural connections (involving nicotinic synapses) are needed to generate the CMMC. In future, other characteristics of the CMMC can be explored in detail using GECIs, such as the observations that 5-HT antagonists (acting at 5-HT1A, 5-HT3, 5-HT4 and 5-HT7 receptors) inhibit or block the CMMC contraction (Heredia et al., 2009, 2013; Dickson et al., 2010b; Smith and Gershon, 2015; Smith et al., 2015; Yu et al., 2015). Although the CMMC contraction increases in frequency following the inhibition of nitric oxide synthesis, it doesn’t propagate effectively down the colon (Heredia et al., 2010). The relative importance and contribution of cellular activity of cholinergic, serotonergic and nitric oxide neurons can be assessed more effectively in mice in which GCaMP3 is expressed in specific cells.

### New Analyses to Extract Information from Intact Segments of Gut

A formidable challenge in monitoring gut neural activity is the ongoing distortion of the tissue generated by spontaneous movements of the colon. Movement compensation is especially critical to monitor in the gut since the ganglionic distortion may be a stimulus to activate certain classes of neurons (Spencer et al., 2003; Mazzuoli and Schemann, 2012). Others have attempted to circumvent the gut movement problem by a number of ways including: (i) recording Ca^2+^ activity from neurons or glial cells either in small preparations of myenteric ganglia from which the longitudinal or circular smooth muscle has been removed; (ii) using cultured neurons or explants of myenteric ganglia; or (iii) using lower non-physiological temperatures or using L-type Ca^2+^ channel blockers or drugs that interfere with actin/myosin interactions (e.g., wortmannin/ML7) to reduce contractile activity (Okamoto et al., 2012; Boesmans et al., 2013a,b, 2015; McClain et al., 2014). Each of these methods invariably introduces some degree of artifact into the responses of cells or tissue.

With the ability to image neurons and glia in undissected tissue with very little background staining, existing analysis methods were inadequate. Especially at low power, the distortions produced by regional contractions and relaxations were never uniform across the FOV. Similarly, the lack of granular background staining (common with Ca^2+^ dyes) meant that the only objects that were distinct enough to track, also had the most changes in brightness due to Ca^2+^ transients. Nuclear differential tracking techniques combined with vector maps appear to offer an elegant solution to extracting data from tissue labeled with GECIs. The event sequence plots allow for detailed examination of activation patterns in relation to the whole FOV, or individual neurons, and can be used to compare the effect of drugs or stimulation patterns in minute detail.

In conclusion, the use of GECIs to study neuronal and glial behavior allows gut preparations to be left fully intact, and allows examination of the complex interactions between both myenteric and submucosal ganglia. As GECIs continue to develop (Akerboom et al., 2012), their excellent signal to noise ratio in a near physiological setting will allow GECIs in *in vivo* experiments to be performed with equal ease. By incorporating the new forms of analysis, the full complexity of neuronal and glial Ca^2+^ events that generate the CMMC or other gut behaviors involving neurons and glia can begin to be elucidated both *in vitro* and *in vivo*.

## Conflict of Interest Statement

The authors declare that the research was conducted in the absence of any commercial or financial relationships that could be construed as a potential conflict of interest.

## Abbreviations

ChAT: Choline acetyl transferase
CMMC: colonic migrating motor complex
ENS: Enteric nervous system
FOV: field of view
GCaMP3: Genetically encoded Ca^2+^ indicator is fused to the calmodulin (CaM) and the M13 domain of a myosin light chain kinase
GECI: Genetically-encoded Ca^2+^ indicator
GFAP: Glial fibrillary acidic protein
nNOS: neuronal nitric oxide synthase
ROI: region of interest
Wnt1: wingless-type MMTV integration site family.
